# Survival after PICU admission: The impact of multiple admissions and complex chronic conditions

**DOI:** 10.1371/journal.pone.0193294

**Published:** 2018-04-05

**Authors:** Håkan Kalzén, Björn Larsson, Staffan Eksborg, Lars Lindberg, Karl Erik Edberg, Claes Frostell

**Affiliations:** 1 Department of Paediatric Anaesthesia, Intensive Care and ECMO services, Astrid Lindgren Children's Hospital, Karolinska Institutet, Karolinska University Hospital (Solna), Stockholm, Sweden; 2 Childhood Cancer Research Unit Q6:05, Department of Women's and Children's Health, Karolinska Institutet, Astrid Lindgren Children's Hospital, Karolinska University Hospital (Solna), Stockholm, Sweden; 3 Department of Anaesthesia and Intensive Care, Children’s Hospital, Paediatric Intensive Care Unit, University Hospital of Lund, Lund, Sweden; 4 Department of Paediatric Intensive Care, The Queen Silvia Children's Hospital, Sahlgrenska University Hospital, Gothenburg, Sweden; 5 Department of Anaesthesia and Intensive Care at Danderyd Hospital, Karolinska Institutet at Danderyd Hospital (KIDS), Stockholm, Sweden; National Yang-Ming University, TAIWAN

## Abstract

**Objective:**

Factors predicting survival over time after pediatric intensive care unit (PICU) admissions are not fully understood. The primary aim of the current study was to investigate whether multiple admissions (MADM) compared to single PICU admissions (SADM) were associated with poor survival over time after being admitted to PICU facilities. Our secondary aim was to investigate if the presence of a complex chronic condition (CCC) would further impair prognosis.

**Design:**

A closed cohort of all children up to 16 years of age admitted to the three PICUs in Sweden between 2008 and 2010 was prospectively collected and followed until 2012, providing survival data for at least one but up to four years of follow-up.

**Setting:**

Three Swedish tertiary referral centers for pediatric intensive care and extracorporeal membrane oxygenation (ECMO) care were used.

**Patients:**

In total, 3,688 Swedish children with 5,019 PICU admissions were included.

**Interventions:**

No interventions were conducted.

**Measurements:**

An extensive data set was recorded, including up to four-year survival information following first PICU admission. The patients were assigned to seven admission diagnostic groups, which were then divided into SADM or MADM groups. The difference in survival over time and mortality rates (MR) and mortality rate ratios (MRR) were calculated. SADM and MADM groups with and without an existing CCC were formed. The difference in survival over time between groups was calculated.

**Main results:**

A highly significant difference in survival over time was noted between SADM and MADM patients (p<0.0001), which was intensified by the presence of a CCC. MADM patients with a CCC had the worst outcome, while SADM patients without a CCC had the best outcome. MADM patients with no CCC demonstrated decreased survival over time compared to SADM patients with a CCC. Survival over time was statistically worsened for patients with MADM compared to SADM for the following admission diagnostic groups: Cardiovascular, Gastrointestinal/Renal, Respiratory, Neurological, and Miscellaneous. The mortality rate (deaths/patient year of follow-up) during the time of follow-up was 0.023 for SADM and 0.062 for MADM patients. The mortality rate ratio (MRR) between these groups was 2.69.

**Conclusion:**

Compared to single admissions, multiple admissions to PICU were associated with a significant decrease in survival over time in some but not all diagnostic groups. Regarding our secondary aim, we found that when the presence of a CCC is factored into the survival analysis, survival over time is further impaired.

## Introduction

For a meaningful description and comparison of pediatric intensive care mortality, scoring systems such as the Paediatric Index of Mortality (PIM) [1, 2] and Pediatric Risk of Mortality (PRISM) [3] have been pioneered, implemented, and improved since the 1990s. At present, PICU mortality is relatively low, [4, 5] and the scoring systems often implemented (PIM and PRISM) are only used for predicting PICU mortality. However, some pediatric patients do die after PICU discharge with or without readmission to the PICU. In this context, settings are poorly described, and reasons for death are not well understood.

A Swedish multicenter cohort-study on PICU admission and long-term outcomes was carried out between 1998 and 2006 [6]. All pediatric admissions to a Swedish Intensive Care Unit (ICU) during a three-year period, including a complete five-year post-admission follow-up, were analyzed. Compared to the Swedish pediatric population without ICU admission, this cohort displayed a persisting 20-fold increase in mortality rate for at least five years post PICU admission. Prior studies have shown that repeated admissions to PICU care and complex chronic/health conditions are associated with increased PICU mortality [7–11]. However, the various factors predicting survival over time after admissions or readmissions to PICU have not previously been investigated. To further describe survival over time after PICU admission and examine the impact of multiple admissions in the different diagnostic groups while adding data on complex chronic conditions, a three-year prospective study and follow-up of the cohort for up to four years was initiated.

## Material and methods

### Ethical approval

The Central Ethical Review Board at the Karolinska Institute approved this nationwide study after consulting with the regional boards at Gothenburg and Lund [Dnr 02–483]. For this cohort, extended ethical approvals were sought and subsequently granted [Dnr 2007/1073-32, Dnr 2008/39-32, KI-Dnr 2009/1295-32. KI Dnr 2016 /2274-32].

### Study design and population

A national, prospective, closed cohort was set up between January 1, 2008 and December 31, 2010. All pediatric patients up to 16 years of age with Swedish 10-digit personal identity numbers admitted to one of the three centers were included in the study. The first noted admission during the study was defined as the index admission. Survival within the cohort after PICU index admission was checked on January 1, 2012, resulting in an assessment after at least one but up to four years post index admission.

### Participating hospitals

During the study period, specialized PICU care was carried out at three locations in Sweden. These included The University Hospital of Lund, The Queen Silvia Children's Hospital at Sahlgrenska University Hospital of Gothenburg, and the PICU and ECMO Centre's of Astrid Lindgrens Children's Hospital at Karolinska University Hospital in Stockholm.

### Patient groups

The main ICD-10 diagnosis stating the reason for index PICU admission was used to assign each patient to one of seven main admission diagnostic groups. These groups were compiled applying the uniform diagnostic coding system used in the Australia and New Zealand Paediatric Intensive Care (ANZPIC) Registry [12]. All patients had one or multiple valid ICD-10 diagnoses registered during their PICU admission.

The diagnostic groups consisted of Injury (Inj), Neurological (Neuro), Postoperative (Post Op), Cardiovascular (CVS), Gastrointestinal/Renal (GI), Respiratory (Resp), and Miscellaneous (Misc). The Misc group included sepsis, post-cardiac arrest, malignancies, endocrine disorders, and allergic reactions in accordance with the recommendations for the ANZPIC registry. Adjustments had to be made for the retrospective nature of the coding; for example, the ANZPIC registry group “Postoperative Cardiovascular” had to be included in the CVS group as it was not possible to differentiate post-operative admissions from other admissions with CVS diagnoses. The diagnoses Postoperative ENT/Thoracic, Postoperative Neuro, and Postoperative Other were all included in one group termed Postoperative (Post Op). Patients were then assigned into two groups depending on the presence of a single admission (SADM) or multiple admissions (MADM) to the PICU. Patients with more than one admission were assigned to early or late readmission groups in conjunction with earlier studies [7, 8]. Early readmission was defined as being readmitted within 48 hours of discharge from PICU, while late readmission was defined as being readmitted after 48 hours post PICU discharge. In accordance with the praxis of European Medicines Agency (EMA) for children [13], five different age groups were also formed: 0–2 days, 3–28 days, >28 days–2 years, >2–12 years, and >12–16 years. The age-group affiliation for each patient was decided by patient age on index admission.

When finalizing the study, we were made aware of the possible impact of complex chronic conditions (CCC) in the PICU population [10]. We therefore added data regarding the presence of a CCC and CCC subcategories into the dataset. The updated list of complex chronic conditions version 2 (CCCv2) with 10 subcategories was used. No patient was assigned to the 11^th^ group, “Premature and Neonatal.” Complex chronic conditions (CCCs) were initially defined by Feudtner [14], further explored in the PICU population by Edwards [10] and updated to the CCCv2 by Feudtner [15]. Combining the presence of a CCC with sorting by reason for PICU admission according to the ANZPIC system, allowed us to describe the data related to underlying chronic morbidity and permitted us to indicate which acute event made PICU care necessary.

### Data collection and validation

For each patient, the following data were collected: personal identity number, PICU admission diagnosis (ICD-10), PIM2, and time and source of admission and discharge. All admissions were checked for the presence of a CCC and a CCC subcategory. If more than one CCC was present, the first noted was used for CCC subcategory classification. The PIM score was verified through an extensive check of data validity by two experienced pediatric intensivists. All admissions were checked for accuracy regarding the time of admission and discharge. After extensive verification of the admission data, 449 temporary so-called “reserve numbers” could be converted to valid personal identity numbers, and 51 non-Swedish citizens were identified and excluded from the study. The personal identity numbers of the patients in the cohort were sent to the Swedish File of National Registration [16] to obtain survival data. The Swedish File of National Registration keeps a governmental-controlled registry of all Swedish citizens, including date of birth and date of death. PICU mortality risk and standardized mortality risk (SMR) were calculated using the PIM2 (2003) model [2]. The Swedish Intensive Care Registry (SIR) [17] has continuously collected admission data and subsequent PIM scores for all Swedish PICU patients since 2007. Because this process was under implementation during the time of this study, data for our investigation were collected directly from each PICU. Complete dataset supported in the S4 Table.

## Statistics

The PICU mortality for each diagnostic, age, or admission group was calculated. Mortality outside the PICU was calculated for the SADM and MADM groups respectively. Long-term survival was expressed as Kaplan-Meier (KM) curves for the single or multiple admission groups and, for the four groups, single or multiple admission with or without a CCC present. Censoring was carried out by January 1, 2012 or at the time of death. Differences between curves and the hazard ratio were examined by using both Log-rank (Mantel-Cox) and Gehan-Breslow-Wilcoxon tests. Likewise, differences between KM curves of single or multiple admissions for the diagnostic subgroups were evaluated. Differences were considered statistically significant when p<0.05; for multi-group comparison of the curves of SADM and MADM with or without CCC, p<0.0083 was considered statistically significant after Bonferroni correction (K = 6). Mortality rates (MR) for the different admission diagnostic groups were calculated (total number of deaths during time of follow-up divided by total accumulated person-time during follow-up), expressed as deaths per year of follow-up time. Patient follow-up time was accumulated from start of index admission to censoring. Mortality rate ratios (MRR) between admission group mortality rates were calculated (MR SADM / MR MADM).

The PICU follow-up data, KM curves, MR, and MRR have been illustrated. Descriptive statistics, curves, and survival calculations were carried out using MS Excel (Microsoft Corporation, Redmond, Washington, USA) and GraphPad prism 5.4 (GraphPad Software Inc. San Diego, USA). Data are given as median values and inter quartile range (IQR).

## Results

### Patient demographics

During the inclusion period of 36 months, 5,019 admissions were made by 3,688 individuals with complete personal identity numbers. A majority of the patients, 79% (n = 2,909) had only one PICU admission, SADM, while 21% (n = 779) of the patients had two or more admissions, MADM. This yielding a total of 2,110 (42% of total admissions). The median length of stay (LOS) was 1.3 days (IQR 0.8–3.7 days), and 28.1% of all admissions had a LOS of more than three days. More admissions were made by males (56.8%) than females (43.2%), and the same proportions of males (56.9%) and females (43.1%) were seen among individuals admitted.

Of the admitted patients, 196 Swedish children had incomplete personal identity numbers or reserve numbers and could not be identified in the Swedish File of National Registration. These children were subsequently lost to follow-up (5.3%).

### Admission diagnostic and age groups

The overall distribution of diagnostic groups at admission was CVS 39%, Resp 20%, Misc 14%, GI 10%, Neuro 10%, Inj 5%, and Post Op 2%. Groups Inj and Post Op combined included 279 admissions.

The median age among patients was 8.7 months. There were 304 admissions (n = 288) of patients between 0 and 2 days of age, 754 admissions (n = 805) from over 2 days up to 2 months of age and 2208 admissions (n = 1195) from over 2 months up to 2 years of age. There were only 400 admissions (n = 332) over 12 years of age. Age groups, LOS, and PICU mortality are presented in S1 Table.

### PICU mortality, SADM, MADM, and CCC survival

**Fig 1:**
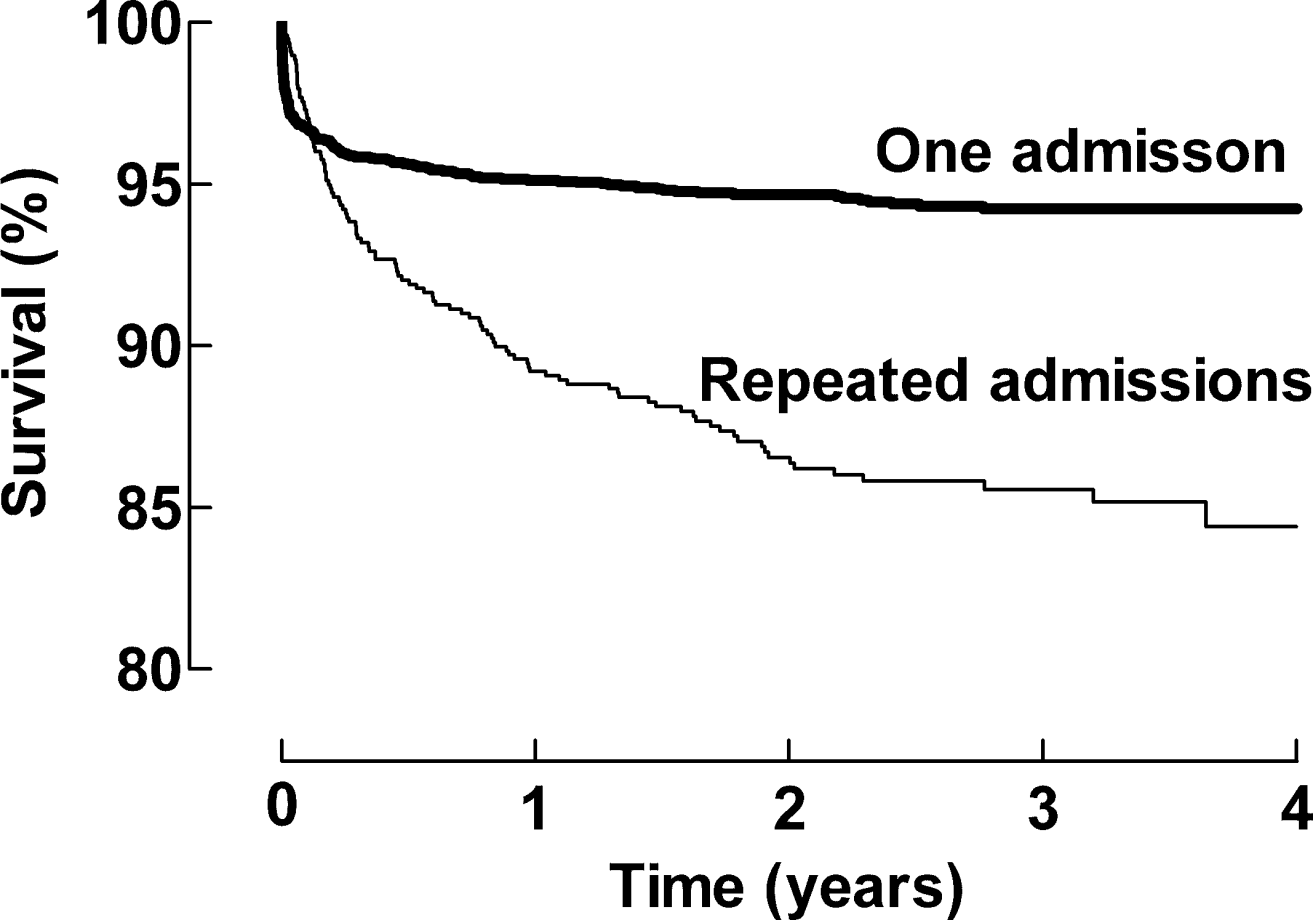
Cumulative survival for the single and multiple admission groups. Statistically significant differences between curves are as follows: Hazard Ratio 3.28; 95% CI 2.43–4.44, Log-rank (Mantel-Cox) or Gehan-Breslow-Wilcoxon Test, both p<0.0001.

The PICU mortality for the cohort was 2.8% (140 deaths in 5,019 admissions). In the diagnostic groups, the PICU mortality was as follows: Misc 8.9%, Neuro 4.5%, Resp 3.2%, GI 2.7%, CVS 2.1%, Inj 1.8%, and Post Op 0.0%. The group of SADM patients (n = 2,909) had a PICU mortality of 3.0% (n = 88), while the group with multiple admissions (n = 779) had a mortality of 6.7% (n = 52) on subsequent admissions. A significant difference was also seen in survival over time between single and multiple admissions groups, Fig 1.

**Fig 2:**
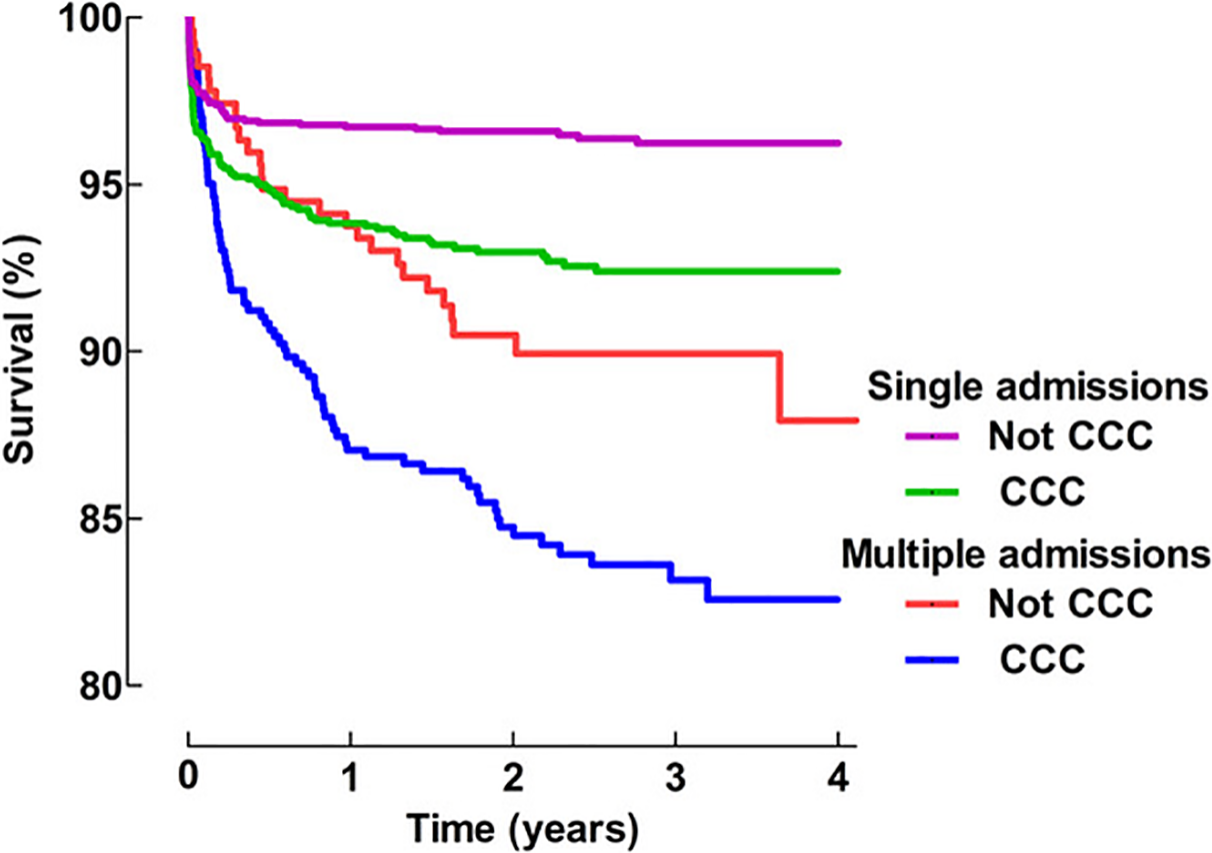
K-M curves for patients with single admissions (SADM) and multiple admissions (MADM) with or without a complex chronic condition (CCC).

A CCC was present in 46.8% (1,728 of 3,688) of the patients. In the group of MADM patients with CCC, 16.2% (82 of 507) of the patients died, which represented 75.2% (n = 90) of all the deaths in the MADM group. In the group of SADM with a CCC, 7.5% (92 of 1,221) of the patients died, which represented 57.5% (n = 160) of all deaths in the SADM group. The cumulative survival for SADM and MADM patient groups with or without a CCC is presented in Fig 2; p-values are provided in Table 1.

**Table 1:** p-values for comparison between patients with single (SADM) or multiple (MADM) admissions with or without a complex chronic condition (CCC).

Subgroup	* *	Subgroup	p-values (Log-rank (Mantel-Cox) Test)	p-values (Gehan-Breslow-Wicoxon Test)
MADM + CCC	*vs*	MADM Not CCC	0.0208	0.0166
MADM + CCC	*vs*	SADM + CCC	<0.0001	<0.0001
MADM + CCC	*vs*	SADM Not CCC	<0.0001	<0.0001
MADM Not CCC	*vs*	SADM + CCC	0.1674	0.2982
MADM Not CCC	*vs*	SADM Not CCC	<0.0001	<0.0001
SADM + CCC	*vs*	SADM Not CCC	<0.0001	<0.0001

Values of p<0.0083 were considered statistically significant after Bonferroni correction (K = 6).

### SADM, MADM and MR for admission diagnostic groups

**Fig 3:**
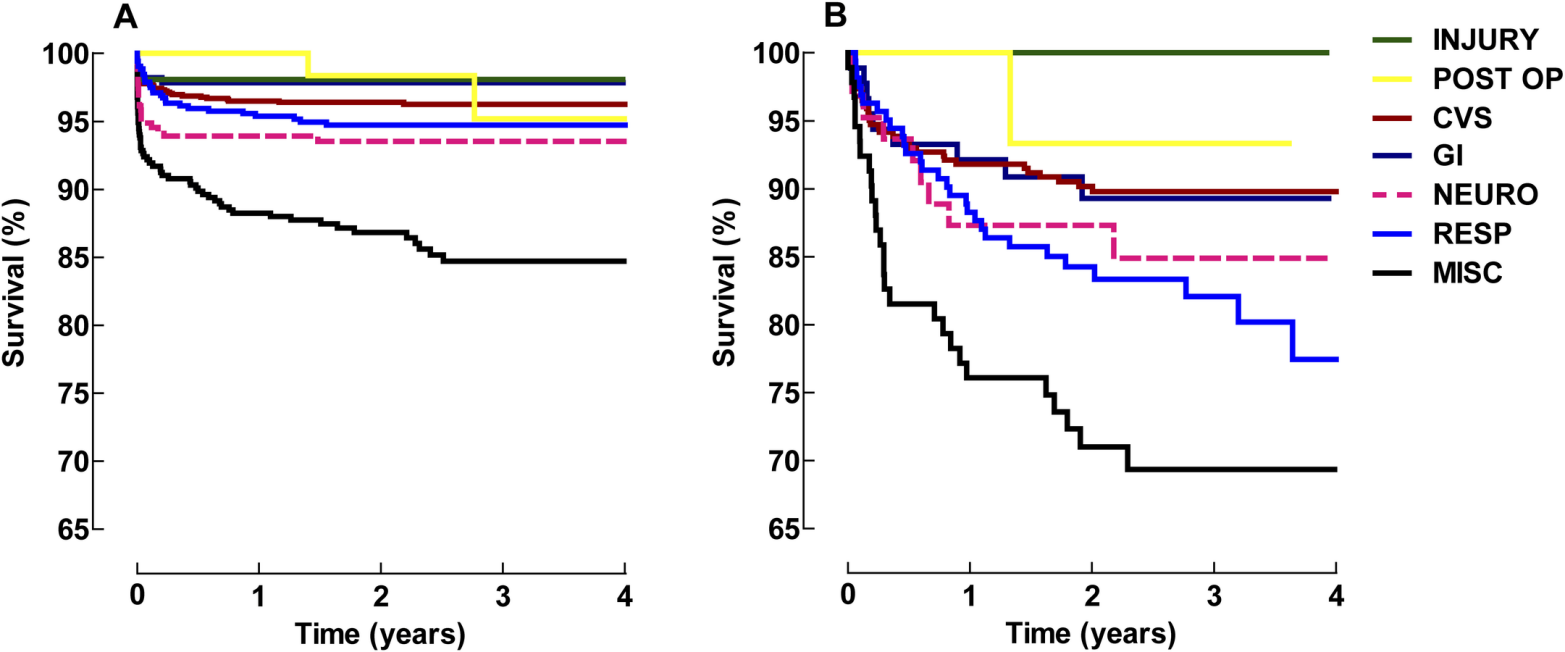
**Kaplan-Meier plots showing diagnostic group survival on single admission (a) and multiple admissions (b).** There was a difference (p<0.001) between single and multiple admissions in all of the diagnostic groups except Inj and Post Op. Neuro displayed a p = 0.04. All p-values are presented in S2 Table.

The impact of SADM and MADM was explored in detail for the diagnostic groups. The results are presented in Fig 3A and 3B.

### PICU mortality, mortality rate, and mortality rate ratio

Total patient follow-up time during the study was 8,688 patient years, accumulated from the start of each index admission. Median follow-up time was 2.6 years (IQR 1.65–3.25) for patients with single admissions and 2.5 years (IQR 1.6–3.3) for patients with multiple admissions. The total mortality in the cohort during the time of the study was 269 individuals and corresponded to a MR of 0.023 in the single admission group and 0.062 in the multiple admissions group giving a MRR value of 2.69. MR and MRR for all diagnostic groups presented in Table 2.

**Table 2:** PICU mortality and mortality rate (MR) with mortality rate ratio (MRR) for single (SADM) and multiple (MADM) admissions groups, depending on admission diagnostic group.

**Single admission**								
ANZPIC Diagnostic group	**All groups**	**CVS**	**GI**	**Injury**	**Neuro**	**Post op**	**Resp**	**Misc**
Numbers of patients	2,909	1,087	279	209	312	70	518	434
PICU mortality n (%)	88 (3.0)	22 (2.0)	5 (1.8)	4 (1.9)	13 (4.2)	0 (0.0)	11 (2.1)	33 (7.6)
Mortality rate Deaths/person years	0.023	0.015	0.009	0.008	0.027	0.011	0.021	0.066
**Multiple admission**								
ANZPIC Diagnostic group	**All groups**	**CVS**	**GI**	**Injury**	**Neuro**	**Post op**	**Resp**	**Misc**
Numbers of patients	779	342	89	15	63	15	163	92
PICU mortality n (%)	52 (6.7)	19 (5.6)	5 (5.6)	0 (0.0)	3 (4.8)	0 (0.0)	11 (6.7)	11 (12)
Mortality rate Deaths/person years	0.062	0.039	0.042	0.000	0.191	0.029	0.107	0.141
**Mortality Rate Ratio**	**2.69**	**2.59**	**4.78**	**0.00**	**7.07**	**2.50**	**5.07**	**2.16**

n = number of patients in group, CVS = Cardiovascular, GI = Gastrointestinal/Renal, Neuro = Neurological, Post Op = Postoperative, Resp = Respiratory, and Misc = Miscellaneous. MR = mortality rate (total number of deaths during time of follow-up divided by total accumulated person-time during follow-up), expressed as deaths per person-years of follow-up time. MRR = mortality rate ratio (MR SADM / MR MADM).

### SADM and MADM with and without CCC in subcategories

To conduct a more in-depth analysis of subgroups, the mortality in the different diagnostic subgroups with and without CCC is presented in Tables 3 and 4 below.

**Table 3:** MADM and SADM patients without CCC and deaths in admission diagnostic groups.

	Patients with Multiple Admissions		Patients with a Single Admission	
	All	Deceased	All	Deceased
Numbers of patients	272	27 (9.9%)	1,688	68 (4.0%)
Median age years (mean)*	0.5 (3.0)	0.4 (3.4)	0.4 (3.2)	1.3 (4.5)
Male sex (% of total)	172 (63%)	19 (11%)	1,156 (57.9%)	40 (3.6%)
ANZPIC diagnostic groups				
Cardiovascular	41	4 (9.7%)	319	12 (3.7%)
Respiratory	93	11 (11.8%)	412	15 (3.6%)
Miscellaneous	35	4 (11.4)	207	19 (9.2%)
Neurological	36	3 (8.3%)	252	11(4.3%)
Gastrointestinal/Renal	43	5 (11.6%)	226	5 (2.2%)
Postoperative	10	0	67	2 (3.0%)
Injury	14	0	205	4 (1.9%)

* Both median and mean age are presented to illustrate skewed distribution.

**Table 4:** MADM and SADM patients with CCC and deaths in ANZPIC admission diagnostic groups and CCC subcategories.

	Patients with Multiple Admissions and a Complex Chronic Condition		Patients with a Single Admission and a Complex Chronic Condition	
	All	Deceased	All	Deceased
Number of patients	507	82 (16.2%)	1221	92 (7.5%)
Age years, median (mean)*	0.1 (1.6)	0.3 (2.4)	1.0 (3.5)	0.7 (3.3)
Male sex (% of total)	276 (54.4%)	40 (14.5%)	675 (55.3%)	53 (7.8%)
ANZPIC diagnostic groups				
Cardiovascular	302	30 (9.9%)	768	28 (3.6%)
Respiratory	69	18 (26.0%)	106	12 (11.3%)
Miscellaneous	56	23 (41.0%)	227	42 (18.5%)
Neurological	27	7 (25.9%)	60	9 (15.0%)
Gastrointestinal	47	4 (8.5%)	53	1 (1.9%)
Postoperative	5	1 (20%)	3	0
Injury	1	0	4	0
Complex Chronic Conditions Subcategories	507	82 (16.2%)	1,221	92 (7.5%)
Age years, median (mean)*	0.1 (1.6)	0.3 (2.4)	1.0 (3.5)	0.7 (3.3)
Male sex	276 (54.4%)	40 (14.5%)	675 (5.5%)	53 (7.8%)
Cardiovascular	308	35 (11.4%)	761	29 (3.8%)
Respiratory	21	2 (9.5%)	47	4 (8.5%)
Neuromuscular	34	9 (26.4%)	82	12 (14.6%)
Congenital/genetic	44	6 (13.6%)	84	6 (7.1%)
Oncologic	40	19 (47.5%)	106	29 (25.5%)
Metabolic/endocrine	14	6 (42.8%)	88	8 (9.0%)
Renal	8	2 (25%)	12	1 (8.3%)
Gastrointestinal	24	1 (4.2%)	35	2 (5.7%)
Hematologic/immunologic	5	2 (40%)	3	1 (33.3%)
Miscellaneous**	0	0	3	0

* Both median and mean age are presented to illustrate skewed distribution.

** Miscellaneous includes rheumatologic, orthopedic, and psychiatric conditions.

### Readmission characteristics

Among all admissions, there were 1,331 (26.5%) readmissions, of which 327 (24.6%) were readmissions within 48 hours from the previous discharge. The median time to readmission in this group was 22 hours (IQR: 8–28 hours), of which 1,004 (75.4%) readmissions occurred more than 48 hours after previous discharge with a median time of 48 days (IQR: 11–160 days). The rate of readmission, counting only early readmissions, was 6.5%. PICU readmission mortality was 6.4% (in 21 out of 327 readmissions) in this group and 3.2% (in 31 of 1,004 readmissions) in the late readmission group. The median age was 3.4 months (IQR: 0.5–20 months) in the early group and 8.5 months (IQR: 3.5–28 months) in the late group. In Swedish PICU facilities, (re)admissions are not classified as scheduled or unscheduled. Deaths among MADM patients—according to diagnostic group, early or late readmissions, PICU status, or final PICU stay—are presented in S3 Table.

### Impact of having an increasing number of PICU admissions

When MADM patients were analyzed regarding number of PICU admissions, they demonstrated between two and 14 admissions during the time of study. The majority of patients had two (64.3%) or three (20.5%) admissions. Only nine children in total had more than eight admissions. Details regarding presence of a CCC and mortality in the patients (depending on the number of admissions) are presented in Tables 5 and 6 below.

**Table 5:** Number of admissions among the MADM patients and presence of CCC and death during the study. 2–7 admissions.

Number of admissions	2	3	4	5	6	7
Individuals n (%)*	501 (64.3)	160 (20.5)	55 (7.0)	32 (4.1)	9 (1.2)	7 (0.9)
Complex Chronic Condition n (%)	306 (61)	105 (65.6)	36 (65.5)	20 (62.5)	7 (77.8)	3 (42.9)
Deaths n (%)	61 (12.2)	20 (12.5)	10 (18.2)	9 (45)	2 (22.2)	2 (28.6)
Deaths with Complex Chronic Condition n (%)	41 (67.2)	15 (75)	6 (60)	5 (55.6)	1 (50)	0

n = number of individual patients

* (%) of 779 MADM individuals.

**Table 6:** Number of admissions among the MADM patients and presence of CCC and death during the study. 8–14 admissions.

Number of admissions	8	9	10	11	12	13	14
Individuals n (%)*	6 (0.8)	3 (0.4)	2 (0.3)	1 (0.13)	1 (0.13)	1 (0.13)	1 (0.13)
Complex Chronic Condition n (%)	5 (83.3)	2 (66.7)	2 (100)	1 (100)	1 (100)	1 (100)	1 (100)
Deaths n (%)	2 (33.3)	1 (50)	0	1 (100)	1 (100)	0	1 (100)
Deaths with Complex Chronic Condition n (%)	1 (50)	0	0	1 (100)	1 (100)	0	1 (100)

n = number of individual patients

* (%) of 779 MADM individuals.

### Transfer to PICU from other ICUs and units

In total, 278 admissions to a PICU were documented as originating from an Adult Intensive Care Unit (AICU) in another Swedish hospital, and 655 admissions occurred from another hospital. In the latter case, it was unclear if these transfers were a referral from an ICU, emergency room, or elsewhere.

## Discussion

### Findings

Survival over time (post PICU discharge) is multifaceted but has only been poorly described previously. A better understanding of factors associated with post PICU care mortality could facilitate better allocation of resources, contribute to sharing correct advice and expectations with parents and caregivers, and identify important targets for future research efforts. The present study demonstrates that multiple admissions to a PICU is associated with a significant decrease in survival over time (Fig 1). This finding was true for most but not all diagnostic main groups, which has not been identified prior to this point.

When the presence of a CCC is added to the survival analysis, survival over time is further impaired (Fig 2). The best survival occurred children with SADM and no CCC, and the poorest prognosis was associated with the presence of a CCC and MADM.

When comparing SADM or MADM within the seven ANZPIC groups, two of the groups (Post Op, Inj) showed no significant reduction in survival over time. However, it is possible that a larger scope of material than the data collected here may display a significant difference also for these subgroups.

For both MADM and SADM with CCC, the CCC subcategories cardiovascular and oncology carried the major part of deaths, added together 66% (n = 54) for MADM, and 64% (n = 58) for SADM, respectively (Table 4). Some of the other subcategories such as metabolic/endocrine also had very high mortality (43%) in combination with MADM but did not add as many deaths in absolute numbers (n = 6).

Both MADMs [7–9] and CCCs [10] have been associated with increased PICU mortality. In congruence, patients with MADMs and predominantly CCCs should exhibit poorer long-term outcomes. Of note, our data show that the different ANZPIC admission groups and CCC subcategories show different survival rates over time, depending on the readmission factor. This is an original finding in the present study.

Early (<48h) or late (>48h) readmission type has been extensively discussed in the literature as a possible marker of quality of care and discharge routines from PICU and ICU [7–9, 18–20]. We chose to focus on survival well beyond these shorter time periods. However, our data on early and late readmissions are available in the S3 Table and the Results section under readmission characteristics.

Information on long-term outcome data after PICU care is sparse but important. A smaller study, where 70 of 253 eligible patients completed a three-year follow-up, showed an increase in cumulative mortality from 3.9% before discharge to 10.4% by three years [21]. These numbers are in line with the findings in the present and previous [4] studies and suggests a population risk in the years after PICU care.

There is a slight discrepancy between studies for [7–9] or against [19] adapting readmission as a risk factor for PICU mortality. At present, neither PIM nor PRISM consider readmission to be a risk factor for mortality. However, we note that a discharge policy causing many early readmissions will systematically add to the denominator value when calculating mortality ratio for a specific PICU. This may create difficulties in correctly evaluating and comparing risk factors for mortality over time. Multiple hospital admissions are the factor most strongly associated with pediatric death by one year post hospital discharge [22]. This was found in a retrospective cohort study, where administrative data on close to 700,000 hospitalized subjects under 21 years of age was collected and evaluated. However, the authors of the cohort study did not incorporate analysis of eventual PICU patients in their cohort, but their observations suggest that multiple admissions could be associated with worse long-term survival after PICU care.

In addition, the impact of chronic critical illness (CCI, defined as length of stay >28 days in PICU) on five-year survival post PICU discharge has been studied [23]. It was reported that children with bone marrow transplant or single-ventricle physiology have the worst long-term outcomes. Using this definition for CCI in our data, we could only identify 18 patients in the SADM and 54 patients in the MADM group. Since so few patients (less than 2%) met CCI criteria, we have chosen not to explore this data further.

### Limitations

The present study has several limitations. First, we chose to collect data only from the three existing PICUs in Sweden that, during the study period, were officially designated as a PICU and equipped and staffed accordingly. However, it is known from a previous nationwide study published in 2008 by Gullberg-Kalzén [6] that only about 50% of Swedish children needing ICU care are actually admitted to a PICU. In that study, primarily older children were treated in Adult Intensive Care Units (AICU)s due to geographical location and limitation of resources (PICU beds). The median age of these AICU treated children was 9.5 years; of these patients, 46% were part of the Inj diagnostic group, and 1.9% experienced ICU mortality. In both the previous and the present cohort, CVS was the dominating diagnostic group for admission, accounting for more than a third of admissions. Neuro had similar numbers in both cohorts (about 10%), but Post Op and Inj were both reduced in the present cohort. This clinical situation remains the same in Sweden, and therefore outcome data are only valid for the three PICUs that contributed. Since 2008, the number of patients transferred from AICU to PICU in Sweden, during a 36-month period, has increased from 65 to 278 as described in the present study. Since 2013, an agreement occurred among Swedish intensivists and involved professional societies that, in order to improve PICU referral strategy at a national level, severely ill children should be transferred to a PICU as early as possible [24].

A second limitation is the comparatively small number of patients in the present dataset. A larger dataset would be of great value for making additional analyses. This may be possible in future multicenter studies. A third limitation is the lack of data on decisions related to “Limitation of Medical Therapy” (LOMT) in the PICU or elsewhere. Judgments by physicians exercising LOMT in end-of-life care are legally accepted in Sweden. Therefore, we cannot determine to what extent LOMT influenced the mortality results of the present study.

A fourth limitation involves the lack of data concerning the physical location of death and detailed circumstances of the event when occurring outside the PICU. Of note, in the SADM and MADM groups, 45% (n = 72) and 52% (n = 57) of deaths took place outside a PICU. Collecting that information is of importance and is currently scheduled as a separate, future study.

A fifth limitation is that the true number of MADM patients might be underestimated by about 5% due to the design of the study, as patients admitted early or late to the cohort might have their first or second admission outside the study period. However, this last minor error will not have conveyed support for our conclusion but would sway our data to the contrary.

Finally, our collected data did not enable us to evaluate to what extent a very late readmission (>1 year) diagnostically was correlated to the index PICU admission, and we did not evaluate the robustness of the link between a very late readmission and the increased mortality seen in MADM patients.

### Strengths

In this study, all PICU treated children in a single developed country were included for three consecutive years and were prospectively followed, with only 5.3% of patients lost to follow-up mainly due to the problem of linking a reserve number on admission to the personal identity number of the patient. In addition, a complete dataset was collected for more than 99% of the admissions.

The important field of survival over time post PICU discharge is complex and not well characterized. Long-term follow-up studies are needed but remain a challenge. This study has identified two strong indicators for impaired survival over time post PICU discharge, namely being repeatedly admitted to a PICU and the presence of a CCC.

### Conclusion

Regarding our primary aim, this study reveals significantly decreased survival over time among children with multiple compared to single admissions to PICU. This association was true for five of seven diagnostic groups. Regarding our secondary aim, we found that when the presence of a CCC is added to the survival analysis, survival over time was further impaired. If confirmed by future work, these observations should improve the understanding of long-term outcomes after PICU discharge.

## Supporting information

S1 Table Age groups, length of stay, and mortality. (XLSX)

S2 Table Survival and p-values estimated from the Kaplan-Meier plots of Fig 3A and 3B. (XLSX)

S3 Table Deaths (n = 109) among MADM patients according to diagnostic group and readmission status. Early corresponds to readmission <48 h post-discharge and late corresponds readmission after 48 h. For abbreviations, see Methods.
(XLSX)

S4 Table Complete dataset. Anonymized.
(XLSX)
